# Perioperative dynamic EEG patterns correlated with changes in cognitive subdomains

**DOI:** 10.1016/j.cnp.2026.07.009

**Published:** 2026-07-14

**Authors:** Duygu Aydin, Mariana Thedim, Chiewlin Liew, Gerhard Schneider, Susana Vacas, Matthias Kreuzer

**Affiliations:** a Department of Anesthesiology and Intensive Care, School of Medicine and Health, Technical University of Munich, Ismaninger Str. 22, Munich 81675, Germany; b Department of Anesthesiology, Mass General Brigham, Harvard Medical School, 55 Fruit Street 444GRB, Boston, MA 02114, USA; c Department of Anesthesiology and Perioperative Medicine, University of California Los Angeles, Los Angeles, CA, USA

**Keywords:** Perioperative risk assessment, Electroencephalography, Montreal cognitive assessment, Mini-mental state exam

## Abstract

**Objective:**

Surgery and anesthesia can be associated with postoperative neurological injury, especially in individuals with diminished cognitive reserve. Timely identification of at-risk patients enables deployment of preventive strategies. Despite consistent recommendations, perioperative cognitive risk assessment remains largely overlooked. We explored which dynamic electroencephalogram (EEG) patterns are correlated with cognitive subdomains significantly affected by surgery and anesthesia.

**Methods:**

This was a secondary analysis of two prospective observational studies that included patients undergoing elective laparoscopic abdominal or pelvic surgery under general anesthesia. Cognition was assessed using the Mini-Mental State Exam, 2nd edition (MMSE-2) and the Montreal Cognitive Assessment (MoCA) within one week before and up to 48 h after surgery. Frontal EEG was recorded pre-induction to extubation. Cognitive subdomains and EEG changes were explored using linear regression and accuracy analyses.

**Results:**

Data were obtained from 23 surgical patients (median [IQR] age, 63 [16] years; 52% female). Auditory-verbal delayed recall (MMSE-2, *p* = 0.001 AUC = 0.76 [0.62–0.89]; MoCA, *p* < 0.001 AUC = 0.75 [0.6–0.89]) and processing speed (MMSE-2, p < 0.001 AUC = 0.85 [0.72–0.95]) were significantly affected postoperatively. Delta power at the end of maintenance, changes in theta, alpha power, aperiodic slope, offset were correlated with processing speed changes.

**Conclusion:**

Dynamic EEG patterns were correlated with processing speed changes. Integrating EEG into perioperative risk assessment may contribute to early identification of vulnerability.

**Significance:**

This study demonstrates the correlation between changes in perioperative EEG and cognitive subdomains.

## Highlights

• To identify perioperative neurocognitive disorder risk, cognitive tests are used.
• Correlation of electroencephalography (EEG) & cognitive subdomains was explored.
• Changes in theta, alpha, aperiodic EEG correlated with processing speed changes.

## 1 Introduction

Surgery and anesthesia-related injury can disrupt patients' cognitive trajectories, leading to measurable declines in performance across multiple domains (Evered et al., 2018; Vacas et al., 2022). These complications, termed perioperative neurocognitive disorders (PND), are a public health concern associated with increased morbidity and mortality, including long-term cognitive decline and dementia (Boone et al., 2020; Gou et al., 2021; Sun et al., 2024). Multiple factors are associated with decreased brain resilience to surgery (Thedim and Vacas, 2025). Preoperative cognitive impairment is a well-established risk factor for PND, but remains unrecognized in up to 64% of patients (Kapoor et al., 2022; Weiss et al., 2023). Assessing perioperative cognitive trajectories is crucial for understanding how surgery and anesthesia affect brain health (Thedim and Vacas, 2024). Early identification of these at-risk patients can support integration of tailored perioperative best practices (Scharp et al., 2026). Time and logistical constraints lead anesthesia teams to refrain from integrating cognitive assessments into their perioperative flow, despite recommendations from multiple societies (Deiner et al., 2020). With projected workforce shortages and increasing clinical demands in high-turnover perioperative settings, this care gap is expected to widen (Menezes and Zahalka, 2024). Risk assessment strategies that are easy and quick to implement are necessary to support brain health, guide preventive measures, and enhance overall outcomes.

Perioperative monitoring tools can serve as valuable components of risk stratification (Thedim et al., 2025b). Commercial neuromonitoring systems that calculate one-dimensional “depth-of-anesthesia” indices are becoming increasingly accessible globally. These systems utilize the changes between awake and anesthetized electroencephalogram (EEG) states, specifically the transition from low-amplitude, high-frequency activity in the awake state to high-amplitude, low-frequency dominant features induced by GABA-ergic agents (Gibbs et al., 1937; Kiersey et al., 1951). Beyond tracking anesthetic state transitions, manifold perioperative EEG features have been associated with postoperative cognitive outcomes. Reported associations include spindle-dominance during emergence and post-anesthesia care unit delirium (Hesse et al., 2019), reduced intraoperative low alpha-power and postoperative subsyndromal delirium (Gutierrez et al., 2019), as well as aperiodic features and periodic alpha features in emergence and post-anesthesia care unit delirium (Ostertag et al., 2024). However, integrating multiple EEG metrics into real-time anesthetic management remains challenging in routine clinical practice. Together, these considerations highlight the need for EEG parameters that are readily interpretable, clinically robust, and meaningfully correlated with cognitive outcomes, particularly those that can inform perioperative brain vulnerability without adding undue complexity to intraoperative workflows.

In this exploratory study, we aimed to identify the correlation between perioperative dynamic EEG patterns and changes in cognitive subdomains following surgery.

## 2 Methods

### 2.1 Study design and patient characteristics

This was a secondary data analysis of two prospective observational studies aimed at examining potential mechanisms and brain tissue changes associated with PND (Roy et al., 2025; Thedim et al., 2025a). Briefly, we included patients aged 40 years or older scheduled for elective abdominal, urologic, or gynecological surgery under general anesthesia (Roy et al., 2025). Exclusion criteria included cardiovascular, hepatic, or renal failure, neurologic disease, including history of epilepsy, severe depression, diagnosis of cognitive impairment or dementia, and alcohol or illicit substance use (Roy et al., 2025). All patients received the same general anesthetic plan consisting of induction with propofol and fentanyl, followed by maintenance with sevoflurane. Continuous noninvasive mean arterial pressure was maintained within 20% of each patient's baseline value or above 65 mmHg using vasopressors as needed. No adjunct medications, such as midazolam, ketamine, gabapentinoids, or dexmedetomidine, were administered in the perioperative period. Patients were monitored intraoperatively with a frontal bihemispheric electrode montage using the SEDLine system (Masimo, Irvine, CA) to guide sevoflurane concentration administration and maintain a Patient State Index (PSI) between 25 and 50. Additionally, EEG wave tracing was monitored to avoid patterns consistent with burst suppression. Enrolment for the parent trials occurred between January 2020 and August 2023. This study was conducted in accordance with all regulatory requirements and ethical principles for human subjects' research. The studies were registered with clinicaltrials.gov (NCT04244162 on January 28, 2020, and NCT04566562 on September 22, 2020) after approval by the Institutional Review Board of the University of California, Los Angeles, #19-001597 and #20-001456. All patients provided written consent before enrollment. This investigation adhered to the STROBE checklist for observational studies.

### 2.2 Neurocognitive assessment

The Montreal Cognitive Assessment (MoCA) (Nasreddine et al., 2005) and the Mini-Mental State Exam 2nd edition (MMSE-2) (Folstein et al., 2010) were administered by trained study team members before (within one week) and after surgery (within 48 h). To minimize learning effects, different versions and forms of the cognitive tests were used at different time points. Scores were normalized according to the manufacturer's instructions for age and education. Trained clinical study team members administered the Confusion Assessment Method (Horwitz et al., 1990) for delirium assessment. Delirium was assessed twice daily for up to three days after surgery or until patient discharge, whichever occurred first. The MoCA, which is commonly used for cognitive screening, comprises 30 items that assess visuospatial abilities, attention, abstraction, memory, language, and orientation. These subdomains overlap with the standard version (SV) of the MMSE (Yang et al., 2021). Both tests include delayed recall components targeting auditory-verbal processes and have a maximum score of 30, with higher values reflecting better cognitive function (Yang et al., 2021). Two additional tasks were also performed as part of the MMSE-2 expanded version (EV): a story memory task targeting verbal explicit learning and free recall, and a symbol digit coding task targeting processing speed (Folstein et al., 2010). Considering these additional tasks, the total of the MMSE-2 EV can reach 90 points, increasing the sensitivity to detect mild cognitive impairment (Folstein et al., 2010). The brief version (BV) has a maximum of 16 points and targets orientation, delayed recall, and memory abilities (Folstein et al., 2010).

### 2.3 EEG recording and analysis

EEG recordings with a sampling rate of 178 Hz were obtained from the SEDLine system in *.edf* format and converted to *.mat* using MATLAB R2023b (Mathworks, Natick, MA). For this secondary data analysis, we included patients with complete intraoperative EEG recordings. Team members performing perioperative neurocognitive assessments were blinded to EEG recordings and analysis.

Artifact-free EEG episodes (15 s) during wakefulness were visually identified as previously reported (Thedim et al., 2025a) (baseline) and at the end of maintenance (pre-emergence), i.e., in the last 2 min before the anesthetic agent was discontinued and the emergence period began. End-maintenance EEG was chosen in addition to the baseline, as the association of intraoperative and emergence EEG with cognitive trajectories is well established (Gutierrez et al., 2019; Hesse et al., 2019). Data points with amplitudes above a 100 μV-threshold were excluded as artifacts. Episodes did not include burst suppression or epileptiform activity. All chosen EEG episodes were filtered to 0.5–45 Hz using a Butterworth forward-backward zero-phase bandpass routine.

For each episode, the absolute frequency band-powers in delta (0.5–4 Hz), theta (4–8 Hz), alpha (8–13 Hz), and beta (13–30 Hz) ranges were calculated using Welch's method for power spectral density (PSD) with a resolution of 0.7 Hz. Oscillations in the delta to alpha frequency bands dominate under GABAergic anesthesia. This can be measured on the forehead due to the process of anteriorization, i.e., an increase in the activity of these bands in the anterior areas (Tinker et al., 1977; Feshchenko et al., 2004), while beta oscillations are usually measured when there is arousal, or with paradoxical excitation (Bevan et al., 1997; Brown et al., 2010).

We used frontopolar electrodes in bipolar montage (Fp1-Fp2), as these channels are typically used for standard EEG-based patient monitoring intraoperatively.

The change in frequency band-powers from baseline to the end of maintenance was calculated by subtracting the sum of power at baseline from the sum at the end of maintenance for each frequency band.

Along with band-powers, two aperiodic parameters, offset and slope, were calculated using the fitting-oscillations-and-one-over-f (FOOOF) toolbox (Donoghue et al., 2020). The toolbox fits a model with aperiodic and periodic components to the power spectrum and defines offset as the y-intercept of the fit, providing information about the EEG amplitude, and the slope as the exponent of the aperiodic component. The aperiodic component of the EEG has been shown to track the hypnotic component of anesthesia (Widmann et al., 2025). The following parameters were used for the toolbox: frequency range = 1–40 Hz to avoid filter edges and a maximum number of peaks = 3, minimum peak height = 0.3, and peak width limits = 2–12 as previously used by (Ostertag et al., 2024). The change in aperiodic offset and slope was calculated in the same way as for the band-powers.

### 2.4 Statistical analysis

As this study represents a secondary analysis of prospectively collected observational data, the data analysis and statistical plan were developed after access to the dataset. We used non-parametric tests for group comparisons due to the small sample size. Linear regression was applied as an exploratory approach to assess associations, with results interpreted cautiously given the sample size.

#### 2.4.1 Neurocognitive assessment

To assess which cognitive subdomains were significantly affected by surgery and anesthesia, we compared preoperative and postoperative MoCA and MMSE-2 global scores, and their subdomains. Data are reported as median with interquartile range [IQR] and differences were assessed using the Wilcoxon Rank Sum test. The Rank Sum test was supported by the effect size area under the receiver operating characteristic (AUC) with 10 k-fold bootstrapped 95% confidence intervals (CI) (Hentschke and Stüttgen, 2011), using the MES toolbox in MATLAB (hhentschke/measures-of-effect-size-toolbox, 2025). Changes were considered clinically relevant if AUC > 0.7 or equivalently <0.3 (indicating AUC > 0.7 in the opposite direction) were observed (Mandrekar, 2010; Hosmer et al., 2013) and significant if *p* < 0.05 and 95% CI of AUC values excluded 0.5 (Hentschke and Stüttgen, 2011).

#### 2.4.2 Collinearity

The significantly affected subdomains and versions were then tested for collinearity with the remaining subdomains using the Spearman rank correlation to avoid repetitive analysis of highly correlated variables. Correlation coefficients ρ and *p*-values are reported.

#### 2.4.3 Linear regression

After eliminating highly correlated or non-independent subdomains, linear regression models were calculated to investigate which EEG parameters derived at the end of maintenance correlated with a change in subdomains. *P*-values and goodness-of-fit measures (R^2^) are reported, along with plots showing scores before and after operation against the EEG parameters, highlighting the change in each subdomain.

#### 2.4.4 Accuracy

Due to the small sample size and resulting low goodness-of-fit measures, an accuracy analysis was added to identify favorable and unfavorable trajectories using the correlated EEG parameters from the regression models.

Each possible subdomain change was assessed for its ability to group patients into favorable and unfavorable trajectories based on EEG parameters, and the accuracy value for the current change (=threshold), i.e., how well the patients' EEG parameters were separated, was determined. The optimal threshold was chosen as the value for which the accuracy was closest to 75%.

#### 2.4.5 Group comparisons

Favorable and unfavorable trajectory groups were compared using the optimal thresholds. First, the EEG parameters at the end of maintenance were compared between the groups using the Wilcoxon Rank Sum test and AUC with 95% CI. Additionally, to identify the dynamics of EEG change related to subdomain changes, the change in EEG parameters was compared between the groups using the same statistical approach. All group comparisons were depicted as superplots (Lord et al., 2020), using the MATLAB function *plotSpread* (plot spread points (beeswarm plot*)*, 2025)*.*

## 3 Results

### 3.1 Patient characteristics and neurocognitive assessments

A total of 23 patients (age 63 [16] years, 52% female) with complete EEG recordings were analyzed. Table 1 summarizes the patients' characteristics.

**Table 1:** Characteristics of the patients included in the study. ASA: American Society of Anesthesiologists; GERD: Gastroesophageal reflux disease; IQR: interquartile range.

Patient Characteristics	
Age, years, median [IQR]	63 [16]
Sex, female, number (%)	12 (52)
Education, years, median [IQR]	14 [2]
Self-reported race, number (%)	
White	22 (96)
Asian	1 (4)
Ethnicity, number (%)	
Not Hispanic	15 (65)
Hispanic	8 (35)
Comorbidities, number (%)	
GERD	13 (57)
Hyperlipidemia	13 (57)
Hypertension	12 (52)
Obstructive sleep apnea	8 (35)
Hypothyroidism	5 (22)
Diabetes Mellitus	4 (17)
Valvular disease	3 (13)
Asthma	3 (13)
ASA physical status, number (%)	
2	6 (26)
3	17 (74)
Surgical procedure, number (%)	
Laparoscopic abdominal surgery	15 (65)
Laparoscopic urologic surgery	6 (26)
Laparoscopic gynecological surgery	2 (9)
Anesthesia time, minutes, median [IQR]	202 [98]

The preoperative and postoperative scores of the MoCA and MMSE-2, including cognitive subdomains, were compared (see Table 2). Two patients were not assessed with the MMSE-2 postoperatively and were therefore excluded from the subsequent analyses concerning the MMSE-2 and its subdomains. The MoCA global score and its delayed recall subdomain were significantly lower postoperatively (*p* < 0.001), with both effect sizes being >0.7, indicative of a clinically relevant effect. MMSE-2 EV, its delayed recall and processing speed subdomains, as well as SV and BV, were significantly decreased postoperatively compared with the preoperative period (Table 2). No patient developed postoperative delirium.

**Table 2:** Neurocognitive assessment to identify subdomain and global score changes. AUC: area under the curve; BV: brief version; EV: expanded version; MoCA: Montreal Cognitive Assessment; MMSE-2: Mini Mental State Exam, second edition; SV: standard version.

Variables			*P* value	*AUC value*
	Before surgery **[*n*** **=** **23]**	<48 h after surgery **[n** **=** **23]**		with 95% CI
Global MoCA Visuospatial Naming Attention Language Abstraction Delayed recall Orientation	26 [3] 5 [1] 3 [0] 6 [1] 3 [1] 2 [0] 4 [2] 6 [0]	24 [4] 4 [1] 3 [0] 5 [1] 3 [1] 2 [0] 1 [3] 6 [0]	<0.001 0.048 1.000 0.509 0.527 0.317 <0.001 1.000	0.71 [0.55–0.86] 0.65 [0.49–0.79] 0.50 [0.50–0.50] 0.58 [0.42–0.73] 0.52 [0.37–0.66] 0.47 [0.37–0.58] 0.75 [0.60–0.89] 0.50 [0.50–0.50]
MMSE-2:EV Visuospatial Naming Attention Language Working Memory Delayed recall Orientation Verbal explicit learning / free recall Processing Speed BV SV	56 [12] 1 [0] 2 [0] 5 [2] 6 [0] 3 [0] 3 [1] 10 [1] 11 [10] 14 [4] 16 [2] 29 [4]	49 [14] 1 [0] 2 [0] 3 [3] 6 [0] 3 [1] 1 [2] 10 [0] 12 [8] 11 [4] 14 [2] 26 [4]	<0.001 0.083 1.000 0.007 1.000 0.180 0.001 1.000 0.835 <0.001 0.001 <0.001	0.69 [0.52–0.84] 0.43 [0.33–0.53] 0.50 [0.50–0.50] 0.67 [0.51–0.82] 0.50 [0.40–0.60] 0.55 [0.43–0.67] 0.76 [0.62–0.89] 0.50 [0.50–0.50] 0.50 [0.32–0.67] 0.85 [0.72–0.95] 0.75 [0.60–0.88] 0.73 [0.57–0.87]

### 3.2 Post-hoc analysis of neurocognitive assessments

Since delayed recall, processing speed, SV, and BV are all components of the MMSE-2 EV, their collinearity was tested with Spearman's rank correlation. BV (ρ = 0.98, p < 0.001) and SV (ρ = 0.71, p < 0.001) changes were significantly positively correlated with changes in delayed recall. Processing speed was not significantly correlated with BV (ρ = 0.29, *p* = 0.21), SV (ρ = 0.13, *p* = 0.57), or delayed recall (ρ = 0.28, *p* = 0.22). Moreover, MMSE-2 delayed recall has a narrower range (0–3) than the MoCA delayed recall (0–5). Therefore, the MMSE-2 processing speed and the MoCA delayed recall were included in further analyses assessing EEG correlations with cognitive subdomains.

### 3.3 EEG correlation with cognitive subdomains

**Fig. 1:**
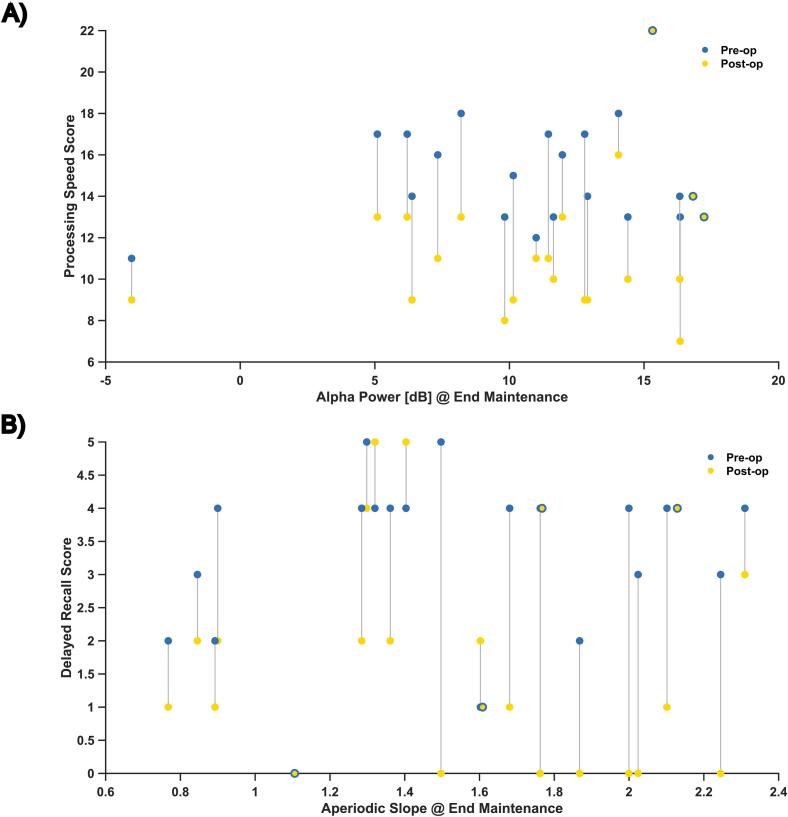
Before and after surgery cognitive subdomain scores, plotted against correlated EEG-parameters. A) Processing speed vs. alpha band-power at the end of maintenance (also called pre-emergence) and B) Delayed recall vs. aperiodic slope of the EEG at the end of maintenance (R^2^ = 0.18, *p* = 0.04). Blue points represent scores before; yellow points represent scores after surgery; each individual patient's scores are connected via a grey line. Blue-encircled yellow points represent patients whose scores did not change postoperatively. (For interpretation of the references to colour in this figure legend, the reader is referred to the web version of this article.)

The delayed recall change from before to after surgery and anesthesia was significantly correlated with the aperiodic slope at the end of maintenance (R^2^ = 0.18, *p* = 0.04), albeit with a small R^2^, while processing speed did not exhibit a significant relationship with any of the assessed EEG parameters (**Table S1**). Therefore, while an aperiodic slope could be used for accuracy analysis, we used the existing literature to select an EEG parameter to calculate an optimal threshold for processing speed. Reese et al. (Reese et al., 2025) recently showed an association between dose-adjusted frontoparietal alpha power and time-sensitive tasks of various tests aggregated under processing speed, indicating that the processing speed of MMSE-2 might also be linearly correlated with alpha power at the end of maintenance. The pre-and postoperative values of both scores, plotted against the chosen EEG parameters, are shown in Fig. 1.

**Fig. 2:**
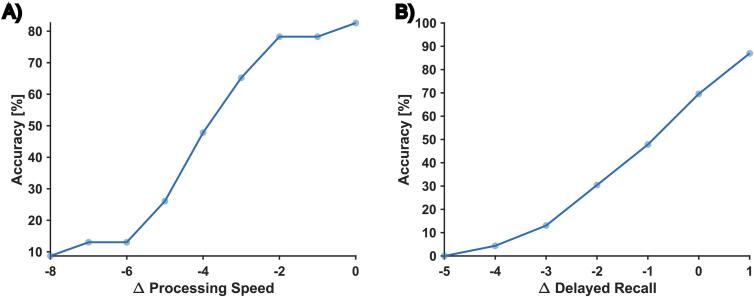
Accuracy analysis of cognitive subdomain change based on the EEG correlates. A) Accuracy of grouping based on processing speed and beta power end-maintenance. B) Accuracy of grouping based on delayed recall and aperiodic slope end maintenance. The optimal threshold was a 2-point decline for processing speed with an accuracy of 78.26%, and zero for delayed recall with an accuracy of 69.57%.

The accuracy of grouping based on alpha band-power for processing speed and aperiodic slope for delayed recall is depicted in Fig. 2. The optimal threshold was a 2-point decline in processing speed with an accuracy of 78.26%, and zero for delayed recall with an accuracy of 69.57%. Patients were grouped into the favorable trajectory if they had ≤2 points of decline in MMSE-2 processing speed, or no decrease in MoCA delayed recall.

### 3.4 Group comparisons

**Fig. 3:**
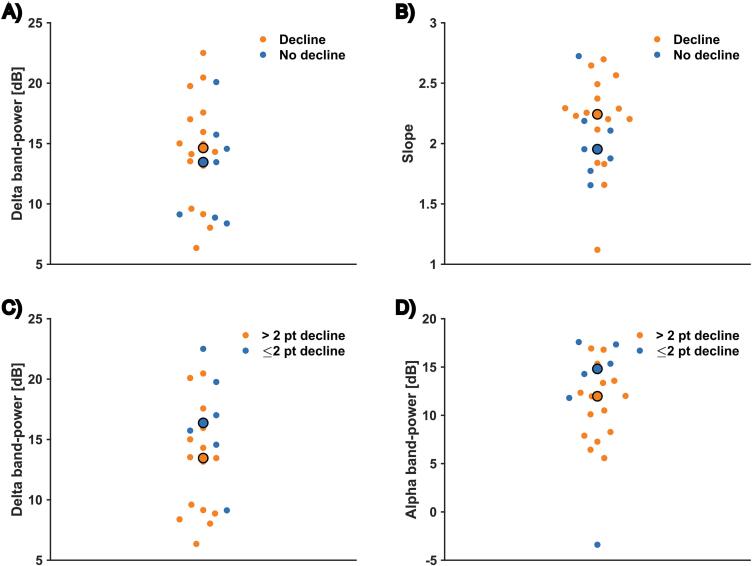
EEG correlates of changes in cognitive subdomain scores at the end of maintenance. A) and B) depict the delta band-power and aperiodic slope of patients experiencing a decline or no decline in delayed recall (p = 0.40, AUC = 0.62 [0.36–0.86] for delta, p = 0.19, AUC = 0.68 [0.40–0.92] for aperiodic slope); C) and D) show delta (p = 0.13, AUC = 0.28 [0.07–0.53]) and alpha (p = 0.20, AUC = 0.31 [0.04–0.63]) band-powers of patients with a processing speed decline less than/equal or greater than 2 points.

Based on the groupings, aperiodic and periodic EEG parameters were compared at the end of maintenance (Fig. 3). No significant differences were observed between the groups for delayed recall (Fig. 3A and B**, Fig. S1, Table S2**). However, if patients showing no change (*n* = 4) were excluded, aperiodic slope (*p* = 0.14, AUC = 0.79 [0.52–0.94]), offset (*p* = 0.21, AUC = 0.75 [0.56–0.96]), and theta power (*p* = 0.17, AUC = 0.77 [0.54–0.89]) were increased in patients with unfavorable trajectories with clinically relevant AUC values (**Fig. S2, Table S3**).

In patients who lost ≤2 points of processing speed postoperatively, only the delta band-power showed a clinically relevant decrease (*p* = 0.13, AUC = 0.28 [0.07–0.53]) (Fig. 3C-D**, Fig. S3, Table S2**).

Next, we investigated whether changes in EEG parameters differed between favorable and unfavorable trajectories. For delayed recall, the change in EEG parameters was not different between the groups, except when patients showing no change were excluded, then the aperiodic slope was lower (p = 0.21, AUC = 0.25 [0.04–0.52]) in patients with favorable trajectories (**Figs. S4–5, Tables S4–5**). In contrast, EEG parameters versus processing speed changes behaved differently.

**Fig. 4:**
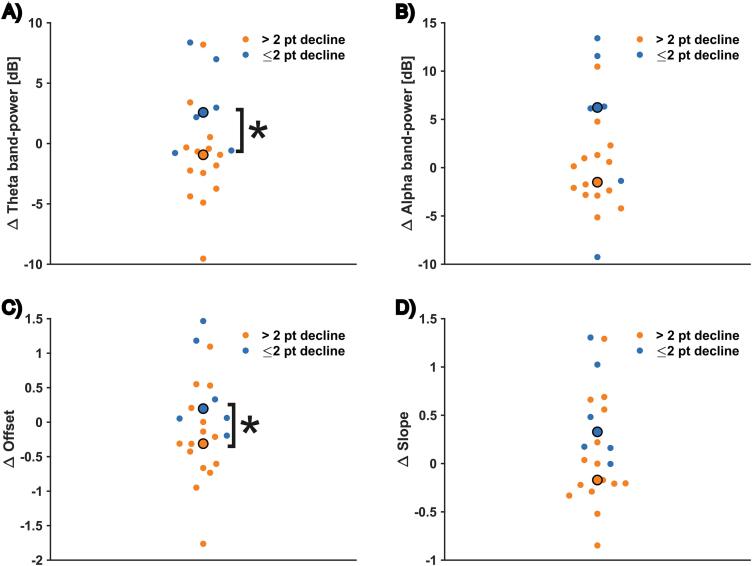
Change in EEG parameters correlated to change in cognitive subdomain scores. A) and C) changes in theta band-power (*p* = 0.047, AUC = 0.21 [0.04–0.43]) and aperiodic offset (*p* = 0.03, AUC = 0.24 [0.07–0.48]) are significantly lower in patients experiencing 2 or more points of decline in processing speed. B) and D) changes in alpha band-power (*p* = 0.11, AUC = 0.27 [0–0.60]) and aperiodic slope (*p* = 0.08, AUC = 0.19 [0.03–0.40]) show a clinically relevant (AUC > 0.7) decline in patients with 2 or more points of decline in processing speed.

Theta band-power (*p* = 0.047, AUC = 0.21 [0.04–0.43]) and aperiodic offset (*p* = 0.03, AUC = 0.24 [0.07–0.48]) changed significantly less in patients who lost >2 points of processing speed; changes in delta (*p* = 0.07, AUC = 0.23 [0.04–0.48]), alpha band-power (*p* = 0.11, AUC = 0.27 [0–0.60]) and aperiodic slope (*p* = 0.08, AUC = 0.19 [0.03–0.40]) were also lower with clinically relevant AUC values (Fig. 4**, Fig. S6, Table S4**).

## 4 Discussion

In this secondary analysis of two prospective observational studies of patients submitted to elective surgery under general anesthesia, we identified delayed recall and processing speed as cognitive subdomains that appear particularly vulnerable to early postoperative changes. Importantly, impairment in these domains correlated with periodic and aperiodic EEG features, suggesting that intraoperative brain activity patterns may offer mechanistic insights into postoperative cognitive vulnerability. These findings support the concept of differential susceptibility of distinct brain networks to surgical and anesthetic stressors, with measurable downstream effects on memory-related cognitive function.

Integrated perioperative approaches that incorporate neuromonitoring tools are increasingly recognized as valuable for identifying patients at increased risk of surgical and anesthetic-related brain injury. EEG monitoring can reveal specific EEG patterns correlated with PND. In clinical settings where preoperative cognitive risk testing cannot be reliably implemented, EEG-based parameters may provide an alternative or complementary method for early risk stratification (Vacas et al., 2022).

We found a positive linear correlation between the aperiodic slope at the end of maintenance and changes in the delayed recall subdomain. A higher positive slope in EEG during emergence has previously been associated with worse postoperative cognitive trajectories (Dragovic et al., 2023), and cohorts with cognitive vulnerability tend to exhibit dominant delta band-power during emergence (Hesse et al., 2019), which can lead to a higher slope. Thus, the correlation of this particular metric with cognitive outcomes is biologically plausible. Although correlations with other cognitive subdomains could not be definitively excluded, particularly given the small sample size, this finding underscores the potential of aperiodic EEG features as mechanistically informative and clinically actionable markers of postoperative cognitive risk.

In contrast, we did not observe significant correlations between selected prefrontal EEG parameters and processing speed. This differs from findings by Reese and colleagues (Reese et al., 2025), who reported associations between dose-adjusted frontoparietal alpha power and aggregated timed processing speed/executive function measures. This discrepancy may be related to the specific EEG epoch analyzed in our study, namely the two minutes preceding the emergence from anesthesia. We speculate that the waning of relative alpha power towards the end of surgery, due to increasing higher frequency activity, may lead to these differences. Alternatively, the limited sample size may have reduced power to detect more modest effects. Nonetheless, alpha power was retained as a reference metric for subsequent accuracy analysis.

To further characterize cognitive trajectories, we performed accuracy analyses to distinguish between favorable and unfavorable outcomes for each subdomain. For delayed recall, we found that the point closest to 75% accuracy in trajectory classification was at zero, suggesting minimal EEG differences between patients with and without postoperative decline when all patients were considered. However, after excluding patients with no postoperative change, group differences emerged with clinically relevant AUC values. This finding suggests that patients classified as “no-change” may represent a particularly resilient subgroup, either due to neurobiological protection against perioperative stressors or because their cognitive trajectories were influenced by factors or time points beyond the 48-h postoperative window assessed here. Future studies should stratify delayed recall performance into decline, no change/stability, and improvement, and evaluate their distinct neurophysiological signatures in larger cohorts. Delta band-power decreased at the end of maintenance in patients with favorable trajectories for processing speed, consistent with the association between increased lower frequency band-power and less favorable cognitive outcomes. Beyond static EEG features, we examined perioperative dynamic changes in EEG parameters, an approach that has not been previously applied to cognitive subdomain-specific outcomes. While changes in delayed recall were not correlated with changes in prefrontal EEG dynamics, reductions in processing speed were correlated with smaller changes in theta band-power and aperiodic offset. Patients with unfavorable processing speed trajectories also exhibited smaller changes in delta and alpha band-power and in aperiodic slope, with meaningful AUC values. From a clinical perspective, theta band-power changes may be particularly attractive as a practical intraoperative indicator, given their relative ease of visual interpretation on raw EEG. In contrast, aperiodic features such as offset and slope are less readily appreciated visually but may be well suited for incorporation into future automated neuromonitoring algorithms.

Our findings build upon prior work linking cognitive subdomains and EEG features that demonstrated that preoperative whole cortex alpha attenuation was greater in patients who made at least one mistake on postoperative attention screening, and that frontal attenuation was associated with delirium severity (Acker et al., 2024). While our analysis focused on frontal EEG signals obtained from a commercial monitoring system, this approach enhances clinical applicability, as two to four-electrode EEG strips are widely used in operating rooms globally.

Despite substantial advances in perioperative care in the last decades, PND remain a critical problem for patients, society, and health care systems. While multiple professional societies recommend routine cognitive assessment for risk stratification, implementation in clinical practice remains underutilized (Deiner et al., 2020). Limited time during preoperative consultations, averaging approximately seven minutes (Compère et al., 2022), likely contributes to this gap, as this duration is insufficient to complete commonly used global cognitive assessments (Kaustov et al., 2022). In contrast, available brief screening tools, such as the Mini-Cog, lack sensitivity to subtle or domain-specific cognitive deficits, particularly in highly educated individuals or those with mild impairments (Vacas et al., 2022). They also provide limited information on baseline cognitive trajectories, constraining risk stratification, mechanistic insight, and the ability to tailor perioperative interventions. Our results align with emerging literature, which emphasizes the need for targeted cognitive screening and intraoperative brain monitoring to identify patients at increased risk for PND. In settings where comprehensive testing is not feasible, focusing on specific cognitive subdomains, such as delayed recall or processing speed, may offer a pragmatic strategy to uncover latent cognitive vulnerabilities in at-risk patients. Overall, focusing on cognitive subdomains may enhance perioperative risk assessment, particularly in fast-paced environments such as preoperative clinics. When cognitive testing is impractical or an assessment cannot be performed, specific EEG patterns may serve as indicators of vulnerability to surgical and anesthetic stress. Perioperative EEG can provide complementary, real-time insight into brain network susceptibility through both periodic and aperiodic features, which can potentially be integrated into commercially available EEG devices (Vacas et al., 2021; Thedim and Vacas, 2024). Ultimately, incorporating subdomain-focused cognitive screening and EEG-derived parameters into perioperative workflows could enable earlier identification of patients at risk for cognitive decline, support individualized anesthetic management, and inform the development of next-generation neuromonitoring algorithms.

This study has several limitations, most notably the small sample size, which limits generalizability and statistical power. Although this constraint makes the observed correlations noteworthy, validation in larger, independent cohorts, including designs specifically tailored to integrate multimodal cognitive assessments with EEG parameters, is warranted. The limited sample size also limited the robustness of the linear regression models, which should be repeated in larger studies incorporating multivariable adjustments. Additionally, missing neurocognitive assessment data and the restriction of EEG analysis to two predefined epochs, baseline and end-of-maintenance, and two prefrontal channels, represent further limitations. These choices were necessary to ensure consistent, time-stamped EEG segments but preclude the evaluation of EEG dynamics during induction and throughout maintenance, as well as the testing of region-specific brain activity correlated to cognitive subdomains. Important global features, such as frontoparietal alpha power, may not be captured by the standard monitoring approach using a frontal montage alone.

Future research should examine whether the findings observed in this study persist across brain regions, as captured by whole-head EEG, and characterize whether they change quantitatively and qualitatively across anesthetic phases.

## 5 Conclusion

In this exploratory study, we demonstrated that perioperative dynamic EEG patterns, particularly theta band-power from baseline to the end of maintenance, are correlated with changes in cognitive subdomains following surgery. These findings support incorporating subdomain-focused cognitive screening and EEG-derived parameters into perioperative brain risk stratification workflows, thereby facilitating the identification of brain vulnerability.

## CRediT authorship contribution statement

**Duygu Aydin:** Conceptualization, Writing – original draft, Writing – review & editing, Data curation, Formal analysis. **Mariana Thedim:** Conceptualization, Writing – original draft, Writing – review & editing, Data curation, Formal analysis. **Chiewlin Liew:** Conceptualization, Project administration, Investigation, Writing – review & editing. **Gerhard Schneider:** Supervision, Visualization, Writing – review & editing. **Susana Vacas:** Conceptualization, Project administration, Supervision, Investigation, Validation, Methodology, Funding acquisition, Writing – original draft, Writing – review & editing. **Matthias Kreuzer:** Conceptualization, Supervision, Validation, Methodology, Writing – original draft, Writing – review & editing.

## Funding

This work was supported by the National Institutes of Health, National Institute of General Medical Sciences K23GM132795 (Vacas). The content is solely the responsibility of the authors and does not necessarily represent the official views of the National Institutes of Health.

## Declaration of competing interest

Drs. Kreuzer and Schneider are co-inventors on several patents related to intraoperative EEG analysis owned by Columbia University and TUM. Matthias Kreuzer received funding from Masimo Corporation, Narcotrend-Gruppe, Medtronic GmbH and Fresenius Kabi Deutschland GmbH for conducting EEG-based training for anesthesiologists. The remaining authors declare no conflicts of interest.
