# Supplementary material for: Perioperative dynamic EEG patterns correlated with changes in cognitive subdomains
Source: Clin Neurophysiol Pract. 2026 Jul 14;11:601–9. doi: 10.1016/j.cnp.2026.07.009 (PMC13393008; doi:10.1016/j.cnp.2026.07.009)

**Supplementary Material**

**Table S1**

|  | Delta  band-power | Theta  band-power | Alpha  band-power | Beta  band-power | Aperiodic slope | Aperiodic offset |
| --- | --- | --- | --- | --- | --- | --- |
| Delayed recall (MoCA) | R^2^ = 0.13,  p = 0.09 | R^2^ = 0.11,  p = 0.12 | R^2^ = 0.04,  p = 0.35 | R^2^ <0.01,  p = 0.86 | R^2^ = 0.18,  p = 0.04 | R^2^ = 0.15,  p = 0.07 |
| Processing speed (MMSE-2) | R^2^ = 0.04,  p = 0.40 | R^2^ = 0.02,  p = 0.54 | R^2^ = 0.03,  p = 0.48 | R^2^ = 0.04,  p = 0.37 | R^2^ <0.01,  p = 0.72 | R^2^ = 0.03,  p = 0.47 |

*Supplementary Table S1: p-values and goodness-of-fit measures (R^2^) of the linear regression models for each combination of periodic and aperiodic EEG parameters vs. delayed recall (MoCA) or processing speed (MMSE-2) subdomains. P- and R^2^-values were rounded to two decimal places.* MoCA: Montreal Cognitive Assessment; MMSE-2: Mini-Mental State Exam, second edition.

**Table S2**

|  | Delta  band-power | Theta  band-power | Alpha  band-power | Beta  band-power | Aperiodic slope | Aperiodic offset |
| --- | --- | --- | --- | --- | --- | --- |
| Delayed recall (MoCA) | p = 0.40,  AUC = 0.62 [0.36-0.86] | p = 0.33,  AUC = 0.63 [0.36-0.89] | p = 0.97,  AUC = 0.51 [0.22-0.79] | p = 0.53,  AUC = 0.41 [0.13-0.71] | p = 0.19,  AUC = 0.68 [0.40-0.92] | p = 0.27,  AUC = 0.65 [0.38-0.89] |
| Processing speed (MMSE-2) | p = 0.13,  AUC = 0.28 [0.07-0.53] | p = 0.37,  AUC = 0.37 [0.11-0.64] | p = 0.20,  AUC = 0.31 [0.04-0.63] | p = 0.56,  AUC = 0.41 [0.13-0.71] | p = 0.41,  AUC = 0.38 [0.12-0.67] | p = 0.26,  AUC = 0.33 [0.08-0.62] |

*Supplementary Table S2: p-and AUC-values with 95% CI from Wilcoxon’s ranksum test and AUC calculations comparing favorable and unfavorable trajectory groups at the end of maintenance. P- and AUC-values were rounded to two decimal places.* MoCA: Montreal Cognitive Assessment; MMSE-2: Mini-Mental State Exam, second edition.

**Table S3**

|  | Delta  band-power | Theta  band-power | Alpha  band-power | Beta  band-power | Aperiodic slope | Aperiodic offset |
| --- | --- | --- | --- | --- | --- | --- |
| Delayed recall (MoCA)  Patients with no change excluded | p = 0.49,  AUC = 0.65 [0.33-0.94] | p = 0.17,  AUC = 0.77 [0.54-0.89] | p = 0.56,  AUC = 0.63 [0.25-0.94] | p = 0.96,  AUC = 0.48 [0.06-0.94] | p = 0.14,  AUC = 0.79 [0.52-0.94] | p = 0.21,  AUC = 0.75 [0.56-0.96] |

*Supplementary Table S3: p-and AUC-values with 95% CI from Wilcoxon’s ranksum test and AUC calculations comparing favorable and unfavorable trajectory groups for delayed recall at the end of maintenance. Patients with no change in delayed recall (n=4) have been excluded. P- and AUC-values were rounded to two decimal places.* MoCA: Montreal Cognitive Assessment.

**Table S4**

|  | Δ Delta  band-power | Δ Theta  band-power | Δ Alpha  band-power | Δ Beta  band-power | Δ Aperiodic slope | Δ Aperiodic offset |
| --- | --- | --- | --- | --- | --- | --- |
| Delayed recall (MoCA) | p = 0.19,  AUC = 0.32 [0.09-0.60] | p = 0.24,  AUC = 0.34 [0.23-0.60] | p = 0.48,  AUC = 0.40 [0.11-0.71] | p = 0.27,  AUC = 0.35 [0.05-0.68] | p = 0.21,  AUC = 0.33 [0.10-0.60] | p = 0.24,  AUC = 0.34 [0.10-0.62] |
| Processing speed (MMSE-2) | p = 0.07,  AUC = 0.23 [0.04-0.48] | p = 0.047,  AUC = 0.21 [0.04-0.43] | p = 0.11,  AUC = 0.27 [0-0.60] | p = 0.26,  AUC = 0.33 [0.07-0.67] | p = 0.08,  AUC = 0.19 [0.03-0.40] | p = 0.03,  AUC = 0.24 [0.07-0.48] |

*Supplementary Table S4: p-and AUC-values with 95% CI from Wilcoxon’s ranksum test and AUC calculations comparing the change in EEG parameters between favorable and unfavorable trajectory groups. P- and AUC-values were rounded to two decimal places, except if the rounding violated p<0.05.* MoCA: Montreal Cognitive Assessment; MMSE-2: Mini-Mental State Exam, second edition.

**Table S5**

|  | Δ Delta  band-power | Δ Theta  band-power | Δ Alpha  band-power | Δ Beta  band-power | Δ Aperiodic slope | Δ Aperiodic offset |
| --- | --- | --- | --- | --- | --- | --- |
| Delayed recall (MoCA)  Patients with no change excluded | p = 0.49,  AUC = 0.65 [0.33-0.94] | p = 0.17,  AUC = 0.77 [0.54-0.89] | p = 0.56,  AUC = 0.63 [0.25-0.94] | p = 0.96,  AUC = 0.48 [0.06-0.94] | p = 0.21,  AUC = 0.25 [0.04-0.52] | p = 0.36,  AUC = 0.31 [0-0.67] |


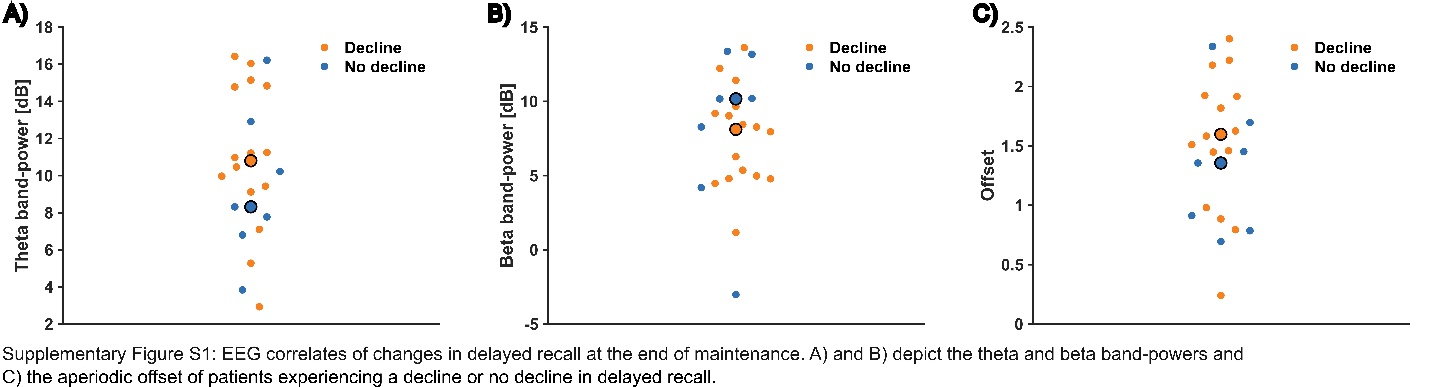
*Supplementary Table S5: p-and AUC-values with 95% CI from Wilcoxon’s ranksum test and AUC calculations comparing favorable and unfavorable trajectory groups for delayed recall. Changes in EEG parameters were compared against changes in delayed recall. Patients with no change in delayed recall (n=4) have been excluded. P- and AUC-values were rounded to two decimal places.* MoCA: Montreal Cognitive Assessment.


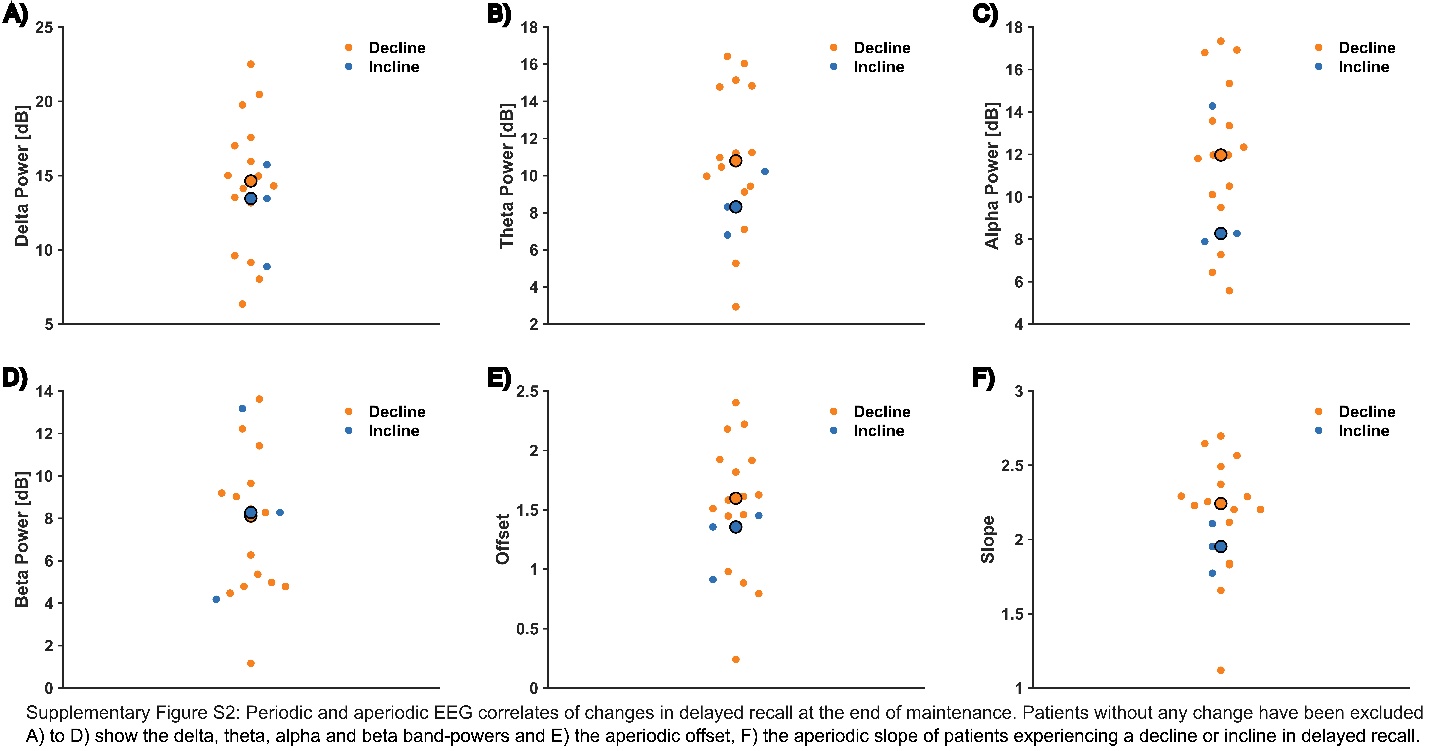


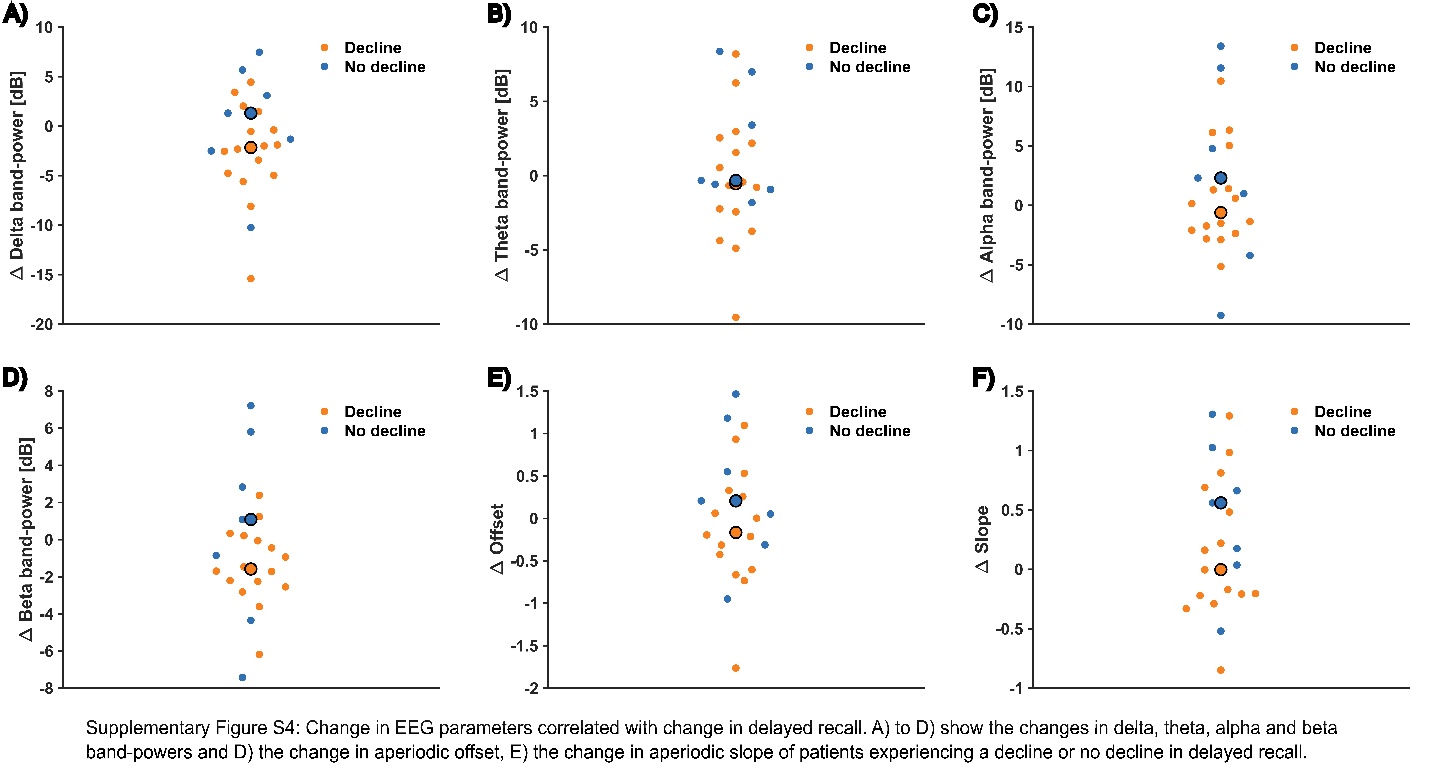

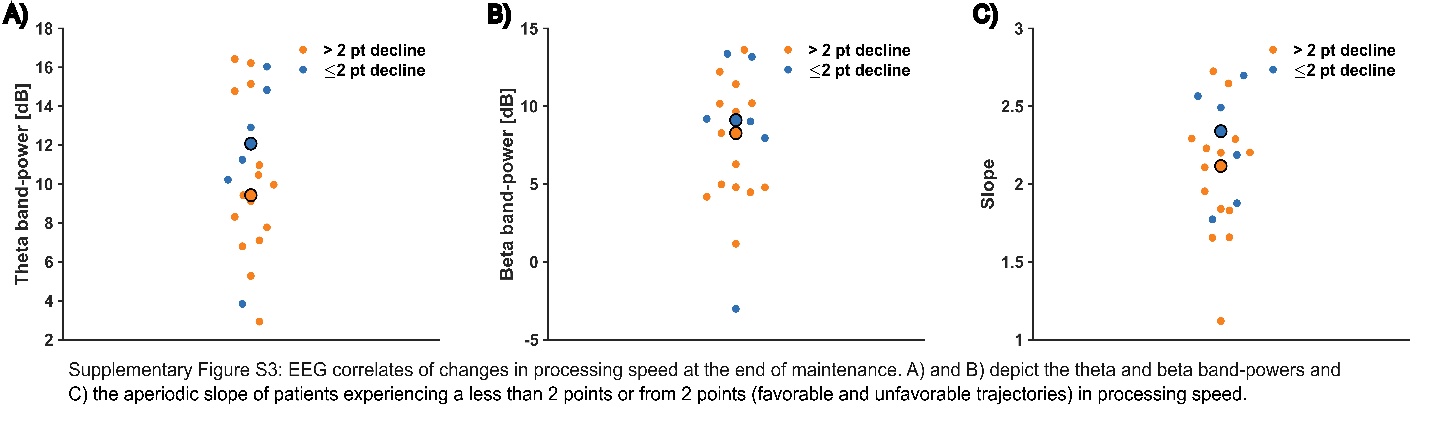


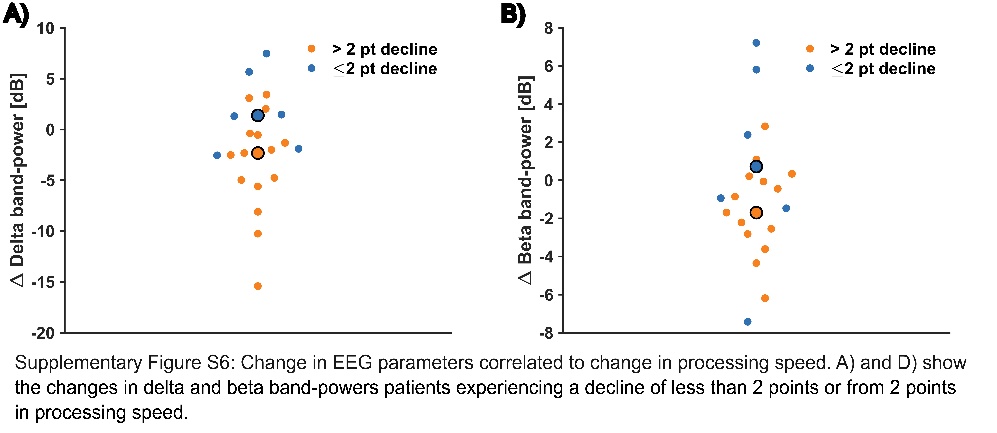

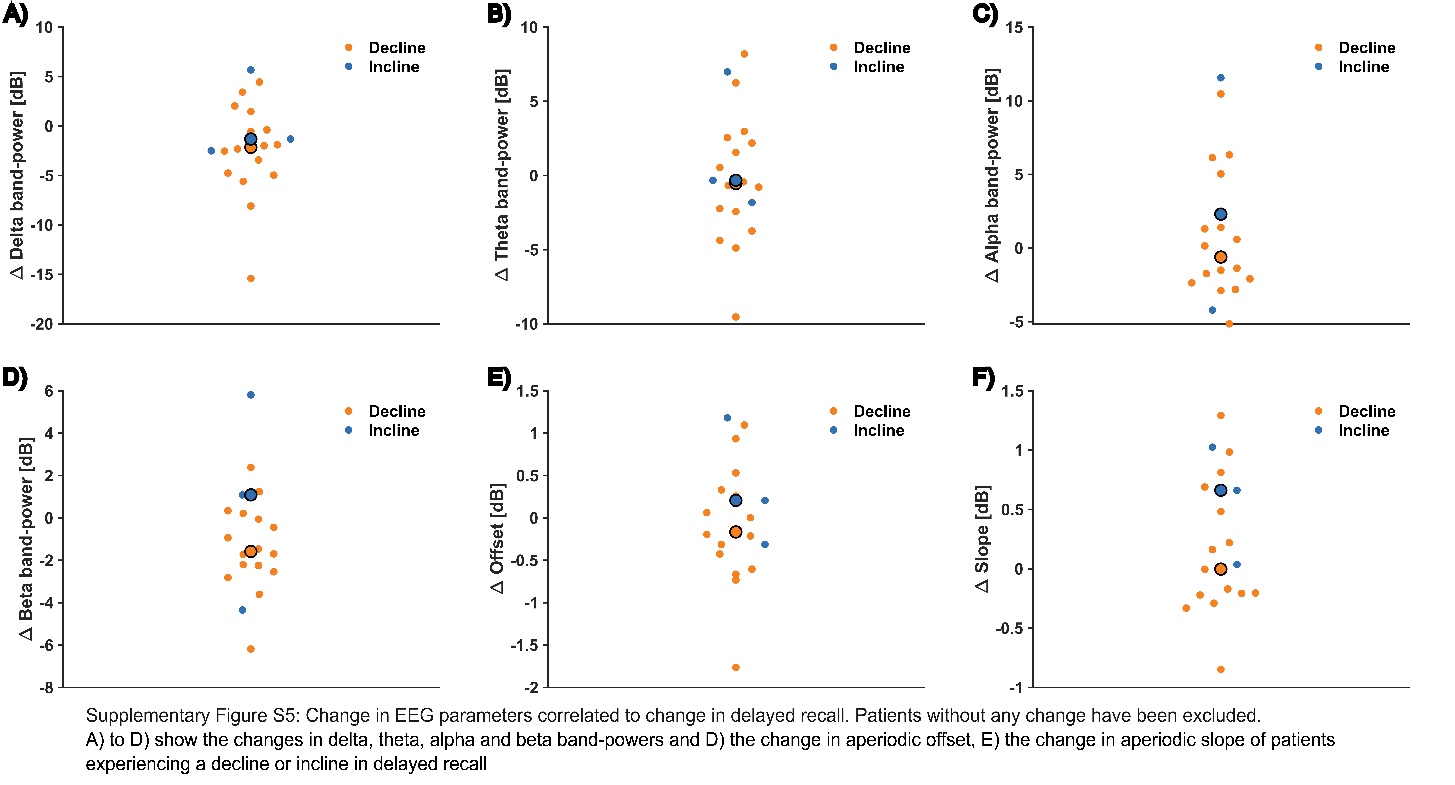

Supplement: Supplementary file 2 — Supplementary material 2 [file mmc2.docx]
